# Establishment of *in vitro* Calibration Curve for ^60^Co-γ-rays Induced Phospho-53BP1 Foci, Rapid Biodosimetry and Initial Triage, and Comparative Evaluations With γH2AX and Cytogenetic Assays

**DOI:** 10.3389/fpubh.2022.845200

**Published:** 2022-08-08

**Authors:** Rajesh Kumar Chaurasia, Kapil B. Shirsath, Utkarsha N. Desai, Nagesh N. Bhat, B. K. Sapra

**Affiliations:** ^1^Radiological Physics and Advisory Division, Bhabha Atomic Research Centre (BARC), Mumbai, India; ^2^Homi Bhabha National Institute (HBNI), Mumbai, India

**Keywords:** 53BP1 foci, γH2AX foci, dicentrics and reciprocal translocations, rapid biodosimetry, radiation induced DNA-DSB detection, colocalization of γH2AX and 53BP1 foci

## Abstract

A rapid and reliable method for biodosimetry of populations exposed to ionizing radiation in the event of an incident or accident is crucial for initial triage and medical attention. DNA-double strand breaks (DSBs) are indicative of radiation exposure, and DSB-repair proteins (53BP1, γH2AX, ATM, etc.) are considered sensitive markers of DSB quantification. Phospho-53BP1 and γH2AX immunofluorescence technique serves as a sensitive, reliable, and reproducible tool for the detection and quantification of DSB-repair proteins, which can be used for biological dose estimations. In this study, dose-response curves were generated for ^60^Co-γ-rays induced phospho-53 Binding Protein 1 (phospho-53BP1) foci at 1, 2, 4, 8, 16, and 24 h, post-irradiation for a dose range of 0.05–4 Gy using fluorescence microscopy. Following ISO recommendations, minimum detection limits (MDLs) were estimated to be 16, 18, 25, 40, 50, and 75 mGy for dose-response curves generated at 1, 2, 4, 8, 16, and 24 h post-irradiation. Colocalization and correlation of phospho-53BP1 and γH2AX were also measured in irradiated peripheral blood lymphocytes (PBLs) to gain dual confirmation. Comparative evaluation of the established curve was made by γH2AX-immunofluorescence, dicentric chromosome assay (DCA), and reciprocal translocation (RT) assays by reconstructing the dose of 6 dose-blinded samples. Coefficients of respective in-house established dose-response curves were employed to reconstruct the blind doses. Estimated doses were within the variation of 4.124%. For lower doses (0.052 Gy), phospho-53BP1 and γH2AX assays gave closer estimates with the variation of −4.1 to + 9% in comparison to cytogenetic assays, where variations were −8.5 to 24%. For higher doses (3 and 4 Gy), both the cytogenetic and immunofluorescence (phospho-53BP1 and γH2AX), assays gave comparable close estimates, with −11.3 to + 14.3% and −10.3 to −13.7%, variations, respectively.

## Introduction

Biodosimetry is known to play a decision-making role to predict prognosis, determine the severity of the case(s), and help subsequent preparation of medical attention, in both planned and unplanned radiation exposures (1). Physical dosimeters like thermoluminescent dosimeters (TLD) and optically stimulated luminescent dosimeters (OSLD) are in practice for dosimetry of occupational radiation workers for long, though, the genuineness of excessive exposures is still ascertained by biodosimetry (2–4). There are various established biodosimetry tools, which can be employed in different scenarios of radiation exposures, for instance, acute (large dose, received in a short period of time) and chronic (sum of small doses received repeatedly, over long durations) radiation exposures. Radiation workers are prone to receive a chronic dose. Acute exposure could be either due to nuclear terrorism (involving, radiation exposure devices, radiation dispersal devices, and improvised nuclear devices) or due to accidents involving nuclear reactor and/or loss or mishandling of radioactive sources. No single biodosimetry tool is known, which can be reliably employed for biodosimetry in all scenarios of radiation exposures. Dicentric chromosome, micronucleus, and translocation assays can be employed for dosimetry of acute exposures, and reciprocal translocation assay is preferred for dosimetry of chronic exposures (5, 6). Dicentric chromosomes are radiation-specific and DCA is considered the gold standard assay for biodosimetry. Micronucleus is not radiation specific, though easy to quantify, and is considered as a method of choice when radiation specificity is not of prime importance (1, 5, 6). With a multi-parametric approach, multiple biological dosimetry methods, mutually supporting the outcomes of each technique, help to draw better scientific conclusions. Hence, it is necessary to establish multiple biological indicators of radiation exposure.

Predominantly, cytogenetic tools like dicentrics and translocations are still in regular practice for dosimetry of occupational radiation workers for regulatory purposes and management of radiological incidents. Dicentrics are categorized as unstable chromosome aberrations. Cells bearing dicentrics progress in the cell cycle to enter mitosis and it has been demonstrated that the number of dicentric chromosomes decreases by about 50% per cell division (7, 8). DCA is the best assay to be employed within a few months after exposure, if the dose is in the range of 0.1–5.0 Gy (whole body exposure) (9). Translocations are categorized as mitotically stable types of chromosome aberrations and represent the method of choice for retrospective biological dosimetry in the dose range of 0.25–4.0 Gy (whole-body exposure) (7, 10). It is a method of choice for past and cumulative dosimetry, up to several years to decades (9). In spite of such remarkable attributes, cytogenetic assays have some limitations for dosimetry of radiological emergencies and/or medical exposures, such as the long and laborious analysis (as cell culturing is obligatory), high MDLs of the assays, and the requirement of experienced technicians to analyze and score aberrations (9, 11).

DSB-repair proteins such as γH2AX, 53BP1, and ATM have gradually sought much attention and are evolving as a potent indicator for rapid biodosimetry. DSBs are frequently induced by radiation exposures; they are created as a result of two single-strand breaks formed on the opposite strands, in the close vicinity, typically in the range of 10–20 base pairs (12, 13). Immediately, after the formation of DSBs, the cell initiates the DNA damage response (DDR), in which signaling molecules like γH2AX, ataxia-telangiectasia-mutated (ATM) protein, ataxia-telangiectasia and rad3-related protein (ATR), and DNA-dependent protein kinase (DNAPKs) are crucial protein players. Minor histone variant, H2AX, gets phosphorylated at ser-139, immediately after DSB formation, in megabase ranges around the DSBs. The 53 Binding Protein 1 (53BP1) is a conserved, check-point protein, which can sense DNA–DSBs, and it gets recruited at the site of DSBs (14–18). Localization of γH2AX and phospho-53BP1 proteins at the site of DSBs can be visualized microscopically as distinct foci in the nuclear region. Certain other DDR proteins like phosphorylated-ATM, Mre11, and NBS1 exhibit similar characteristics and can be explored as an indicator of radiation exposure (19, 20). Phospho-53BP1 and γH2AX assays have emerged as a very sensitive, reliable, and reproducible method for the detection and quantification of DSBs (21). Besides radiological, occupational, and emergency biodosimetry applications, both cytogenetic indicators and phospho-53BP1 and γH2AX assays can serve as reliable tools for clinical applications, such as determination of radio-sensitivity and/or clinical estimation of scattered dose or bone marrow dose in therapeutic and diagnostic applications. In particular, in a few interventional and diagnostic applications, the doses are considerably lower than the MDLs of cytogenetic assays. The added advantage of the possibility of controlled collection of the blood sample after the procedure can further enhance the application of γH2AX and phospho-53BP1 foci assays. These assays have extensively been studied for dosimetry of planned exposures in clinical and non-clinical applications and in human and non-human samples (22–26). Unlike cytogenetic assays, γH2AX and phospho-53BP1 foci assays have not been much explored for biodosimetry of radiological incidences (unplanned exposures) involving human exposures. To apply protein markers (53BP1 and γH2AX foci assay) for biodosimetry, it is essential to highlight some inherent and technical limitations of these markers (27–29). Protein markers are short-lived (hours to days) since they disappear on the completion of DSB repair, contrary to cytogenetic markers that can be detected months and even years after radiation exposure (27). Another limitation of protein markers is the loss of linearity with dose due to overlapping of foci at higher doses (>2 Gy, at 30 min) (28): for accurate measurement of foci, overlapping of foci should not exceed 20% (29). Nevertheless, the sensitivity of protein markers is greater than any other established biodosimetry marker (21).

Biodosimetry Laboratory at Bhabha Atomic Research Center (BARC), the central facility in India, acts as a reference laboratory for the biodosimetry network established in the country. Dose-response curves generated act as a national standard and serve as a reference calibration curve for the other biodosimetry laboratories present in the national network. We are also in the process to extend this network with national and international laboratories.

## Materials and Methods

### Study Design

The study was designed to (1) measure the background levels of phospho-53BP1 foci in the blood sample of 5 volunteers (age range 22–35 y) by immunofluorescence assay, (2) analyze *ex vivo* decay kinetics for the dose points 0, 1, 2, 3, and 4 Gy up to 24 h post-irradiation, (3) generate dose-response curves for ^60^Co-γ-rays (0–4 Gy)-induced phospho-53BP1 foci at 1, 2, 4, 8, 16, and 24 h post-irradiation, (4) estimate MDLs for all 6 dose-response curves, following ISO recommendations (30). Moreover, comparative evaluation of immunofluorescence (phospho-53BP1 and γH2AX) and cytogenetic assays (dicentrics and reciprocal translocations) was performed by reconstructing the dose of 6 dose-blinded samples.

### Ethical Approval, Volunteer Selection, and Blood Collection

The research proposal was approved by the institutional ethical committee (Bhabha Atomic Research Center, Medical Division, BARC). The project was executed following all the ethical guidelines provided. Five healthy volunteers, two females designated as V1 and V2 with ages 22 y and 24 y, and 3 males designated as, V3, V4, and V5 with ages, 25, 31, and 35 y were recruited to this study. None of the volunteers had any history of exposure to ionizing radiation or any other clastogenic agents such as smoking and alcohol consumption. A total of 25 ml blood sample was obtained in heparinized vacutainer tubes from each volunteer by puncturing the cubital vein by an expert phlebotomist.

### Lymphocyte Preparation and Irradiation

Peripheral blood lymphocytes s were prepared from whole-blood sample by density gradient centrifugation using Histopaque-1077 (Sigma-Aldrich, USA) following the manufacturer's protocol with some modifications (31). In short, after 1:1 dilution with HBSS (Gibco Life Technologies, USA), blood samples were slowly and steadily layered on the top of the histopaque in fresh centrifuge tubes. Tubes were gently loaded and centrifuged at 2,500 rpm for 25 min at room temperature. Buffy coat layer was gently withdrawn from each tube and resuspended in RPMI (cell density 1 million/ml; Gibco Life Technologies, USA) without serum after two brief washes with RPMI. Lymphocyte suspensions were aliquoted in fresh centrifuge tubes (0.5 ml/tube) and were irradiated with ^60^C0-γ-rays in the dose range of 0.05–4.0 Gy using Blood Irradiator-2000, manufactured and supplied by the Board of Radiation & Isotope Technology (BRIT), DAE, India. All the irradiations were carried out at the dose rate of 1 Gy/min.

### Immunofluorescence Staining, Imaging, and Scoring of Phospho-53BP1 Foci

Following irradiation, lymphocyte suspensions were kept in ice until reaching the laboratory to reduce the pace of DNA repair. After receipt at the laboratory, cell suspensions were supplemented with 15% FCS (Gibco Life Technologies, USA) and were incubated for various incubation times (described below) in optimum conditions (5% CO_2_, 95% relative humidity, and 37°C temperature). Following incubation, cells were fixed with an equal volume of 4% paraformaldehyde (Gibco Life Technologies, USA; final working concentration of 2%) at 4°C for 30 min. Fixed cells were immunofluorescence-stained for the detection of phospho-53BP1 following our earlier established γH2AX protocol with some modification (32, 33). In short, fixed cell suspensions were spotted on 22 × 22 poly L-lysine (Sigma-Aldrich, USA) coated cover glasses and were allowed to adhere to the cover glasses for 1 h in a humidified chamber. Unattached cells were washed off with 1X PBS. Cells were permeabilized with detergent, 0.5% Triton X-100 (Gibco Life Technologies, USA) in PBS for 5 min and washed 3 times (5 min each) with PBS to remove remnants of detergent completely. Blocking was carried out with 5% FCS for 1 h in a humidified chamber and no washing was given afterward. A total of 50 μl of rabbit anti-phospho 53BP1 human monoclonal IgG (primary antibody; Cell Signaling Technology, Massachusetts, USA) with a dilution of 1:200 (in PBS with 2% FCS) was applied on each cell bearing cover glass and incubated for 1 h in a humidified chamber. Thereafter, unattached antibodies were washed off with 1X PBS, 3 times, 5 min each (the last wash was with PBST, PBS + 0.1% tween-20). Cells were then treated with Texas red-labeled goat anti-rabbit IgG (secondary antibody; Invitrogen, USA) and incubated in a humidified chamber for 1 h. Unattached antibodies were washed off with 1X PBS 3 times 5 min each [last wash was, with PBST, PBS + 0.1% tween-20 (Sigma-Aldrich, USA)]. Cells were air-dried, mounted with DAPI (Invitrogen, USA) with antifade, and sealed with rubber cement after incubation for 10 min.

Mounted slides were loaded on an automated fluorescence microscope, Axioscope Imager M1 (Carl Zeiss) installed with Metafer 4 software, and scanned for the detection and quantification of phospho-53BP1 foci using a Metacyte classifier at 63X magnification. More than 300 cells (in triplicate) were analyzed per dose point, for each volunteer in an automated mode (detection, capturing, and scoring of foci in each lymphocyte nucleus) to estimate foci yield per dose point per individual. Manual verifications were made for a few randomly selected dose points, and no statistically significant differences (*p* < 0.05, *t*-test) were observed in manual vs. automated scorings.

### Background Level of Phospho-53BP1

More than 300 PBLs were analyzed to score the background frequency of phospho-53BP1 foci in each sample (five individuals with triplicate sampling, total 15 samples). In total > 4,500 PBLs were scored.

### Decay Kinetics

The decay kinetics of phospho-53BP1 foci were studied in the blood sample of all 5 volunteers (details mentioned in Section Ethical approval, volunteer selection, and blood collection) for acute doses of 0, 1, 2, 3, and 4 Gy. For each set of experiments (per dose point), isolated PBLs were aliquoted into 6 parts (1 million per aliquot) and incubated in optimum conditions (5% CO_2_, 95% relative humidity, and 37°C temperature) for 1, 2, 4, 8, 16, and 24 h after irradiation. Following the given period of incubation, samples were fixed with 2% PFA and processed by immunofluorescence for the detection and quantification of phospho-53BP1 foci, as described above. Triplicate blood sampling and processing were carried out per dose point, per volunteer, for each incubation time.

### Dose-Response Curve

Dose-response curves were generated with the blood samples of all five volunteers (V1–V5, details described above) after 1, 2, 4, 8, 16, and 24 h of incubation, post-irradiation (dose range 0.05–4 Gy). Irradiation, post-irradiation incubation, immuno-fluorescence staining, scanning, and scoring of phospho-53BP1 foci were carried out as described above.

### Co-staining (Immunofluorescence) of Phospho-53BP1 and γH2AX, DDR Proteins

The same antibodies and procedures mentioned in Section Immunofluorescence staining, imaging, and scoring of phospho-53BP1 foci were employed for the co-staining of phospho-53BP1 and γH2AX proteins (32). Imaging was carried out under 3 filters, blue (DAPI), red (Alexa Fluor 594), and green (FITC 488), for nucleus, phospho-53BP1, and γH2AX, respectively.

### Comparative Validation of the Established Calibration Curve

To validate established ^60^Co-γ-rays-induced phospho-53BP1 dose-response curves, a volunteer (male, age 27 y) was recruited who was not part of the dose-response curve study. Objectives were explained and ethical consent was obtained from him. A total of 24 ml blood sample was withdrawn from him by a phlebotomist and was equally divided into 6 parts (4 ml each) named Z1–Z6. These samples were irradiated with ^60^Co-γ-rays at different blinded doses. Following irradiation, all blinded samples were processed for detection and quantification of γH2AX foci, phospho-53BP1 foci, dicentric chromosome assay (DCA), and reciprocal translocations (RT).

### Immunofluorescence (γH2AX and Phospho-53BP1) Validation of the Established Calibration Curve

Out of 4 ml of blood (of each sample, Z1–Z6), 2 ml was used for the isolation of PBLs for γH2AX and phospho-53BP1 assays. For γH2AX and phospho-53BP1 immuno-fluorescence staining, the same procedure was followed as described above, except that the primary and secondary antibodies were different for γH2AX staining. All blinded samples were fixed and processed for γH2AX and phospho-53BP1 after 1 and 4 h of incubation in optimum conditions (5% CO_2_, 95% relative humidity, and 37°C temperature) post-irradiation. To estimate the yield, more than 300 cells were analyzed for each blinded sample for γH2AX and phospho-53BP1 independently. Doses were reconstructed following coefficients of dose-response curves generated at 1 and 4 h post-irradiation.

### Cytogenetic Validation of the Established Calibration Curve

The remaining 2 ml of blood (of each sample, Z1–Z6) was used for DCA and RT. For detection and quantification of dicentrics and reciprocal translocations, whole blood samples were incubated for 3 h in optimum conditions (5% CO_2_, 95% relative humidity, and 37°C temperature) after irradiation to allow repair to happen and give rise to post-repair events like dicentrics and translocations (34, 35). After incubation, four sets of whole blood cultures were set up for each blinded dose (in total 24 cultures), following our standard protocol (36), optimized as per IAEA and ISO recommendations (9, 30, 37). Cultures were terminated and processed at 52 h of incubation and two slides were prepared per culture. Long-term colcemid (Gibco Life Technologies, USA) treatment (added at 24th h, from culture setup) was given to avoid cells entering into the second cell division cycle (38). Slides were stained with Giemsa (Sigma-Aldrich, USA) for 10 min, rinsed with distilled water, air-dried, and mounted with DPX. Slides were scanned with Axio Imager M1 installed with the software Metafer 4 (Metafer4-V3.9.6) from MetaSystems (Altlusshein, Germany) in a semi-automated mode. Metafer 4 consists of two modules: MSearch to locate metaphases, and Autocapt for automated high-resolution image capturing at 63X magnification. To estimate the yield of dicentrics, more than 500 captured metaphases were analyzed manually per sample, following IAEA and ISO recommendations (9, 37).

Reciprocal chromosomal translocations were analyzed by FISH, using a whole chromosome paint probe (MetaSystems, Germany) for chromosome pairs 1 and 2 (fluorophore FITC for pair 1 and Texas Red for Pair 2) following the manufacturer's protocol with slight modifications (39–41). In brief, slides were first treated with RNase (Thermo Fisher Scientific, USA) and pepsin (Sigma-Aldrich, USA). After that, 16 μl probe (8 + 8, for chromosome pair 1 and 2) was applied to the slides, then covered with glass cover, and sealed with rubber–cement. Denaturation was carried out on a hotplate at 75°C (±1°C) for 3 min. Further, the slides were incubated overnight for hybridization in a humidified chamber at 37°C. After hybridization, rubber cement and coverglasses were removed, followed by a brief rinse in 0.4X SSE at RT. To avoid non-specific binding of the probes, slides were washed with 0.4X SSC at 72°C (±1°C) for 3 min followed by a rinse of 2X SSC containing 0.05% Tween-20 for 2 min. A brief rinse (two times) of distilled water was also given to dissolve and remove salts. Slides were dehydrated with ethanol series (80, 90, and 100%, 1 min each) and air-dried. Finally, slides were mounted in DAPI with antifade and scanned by Axio Imager M1, installed with software, Metafer 4, module ISIS. To estimate the yield of reciprocal translocations, more than 500 captured metaphases were analyzed per sample following IAEA and ISO recommendations (9, 41).

### Statistical Analysis

All experiments were carried out in three sets and the generated data were presented as mean ± standard deviation (SD) or mean ± standard error of mean (SEM). Student's *t*-test was conducted to compare means and determine statistical differences for different doses and time points. The level of significance was set to alpha = 0.05. MDLs were estimated for dose-response curves established at 1, 2, 4, 8, 16, and 24 h incubation after irradiation to ensure estimated doses are significantly higher than 0 at 95% CI. As suggested in ISO standards, 3σ (>99% CL) variation of the background frequency was used to estimate MDLs of all the established calibration curves. Curves were constructed following the least square method.

## Results

### Background Level of Phospho-53BP1

**Figure 1:**
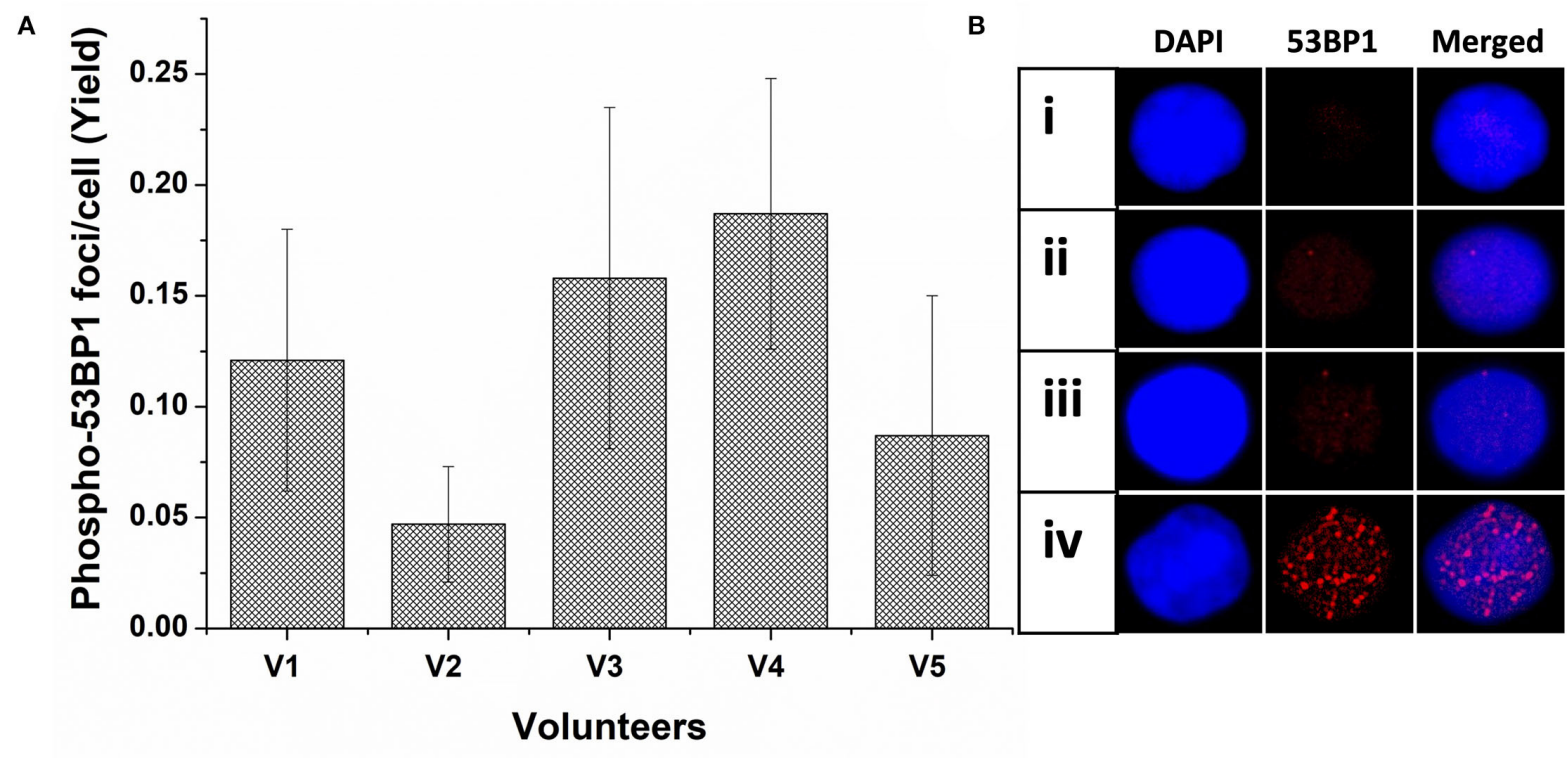
**(A)** Spontaneous frequency of phospho-53BP1 foci in non-stimulated and unirradiated PBLs of five volunteers (V1-V5). Error bars signify SD (5 × 3 i.e., five individuals with triplicate blood sampling). More than 300 PBLs were analyzed for estimating the spontaneous frequency of phospho-53BP1 in each volunteer. **(B)** Representative PBLs with (i) no foci (ii) one focus (iii) two foci (iv) numerous foci (could be apoptotic cell).

As illustrated in Figure 1A, the background level of phospho-53BP1 was measured in the PBLs of 5 volunteers. The mean cumulative yield was found to be 0.12 ± 0.057, varying from 0.047 ± 0.026 to 0.187 ± 0.061.

Among the cells (unirradiated) analyzed, most cells (~91%) were with no foci [Figure 1B(i)] and only a few cells were with foci [~9%, Figure 1B(ii,iii)]. Cells with one focus [~7% cells, Figure 1B(ii)] were predominant in comparison to cells with two foci [~2% Figure 1B(iii)]. None of the cells with ≥ 3 foci/cell were observed. A few cells (<0.6%) were found with numerous, overlapping, and indistinct foci [Figure 1B(iv)], considering them outlier (could be proapoptotic cell), and excluded from the scoring. Morphologically, observed foci were less prominent and smaller in size in comparison to foci induced in radiation-exposed cells.

### Decay Kinetics

**Figure 2:**
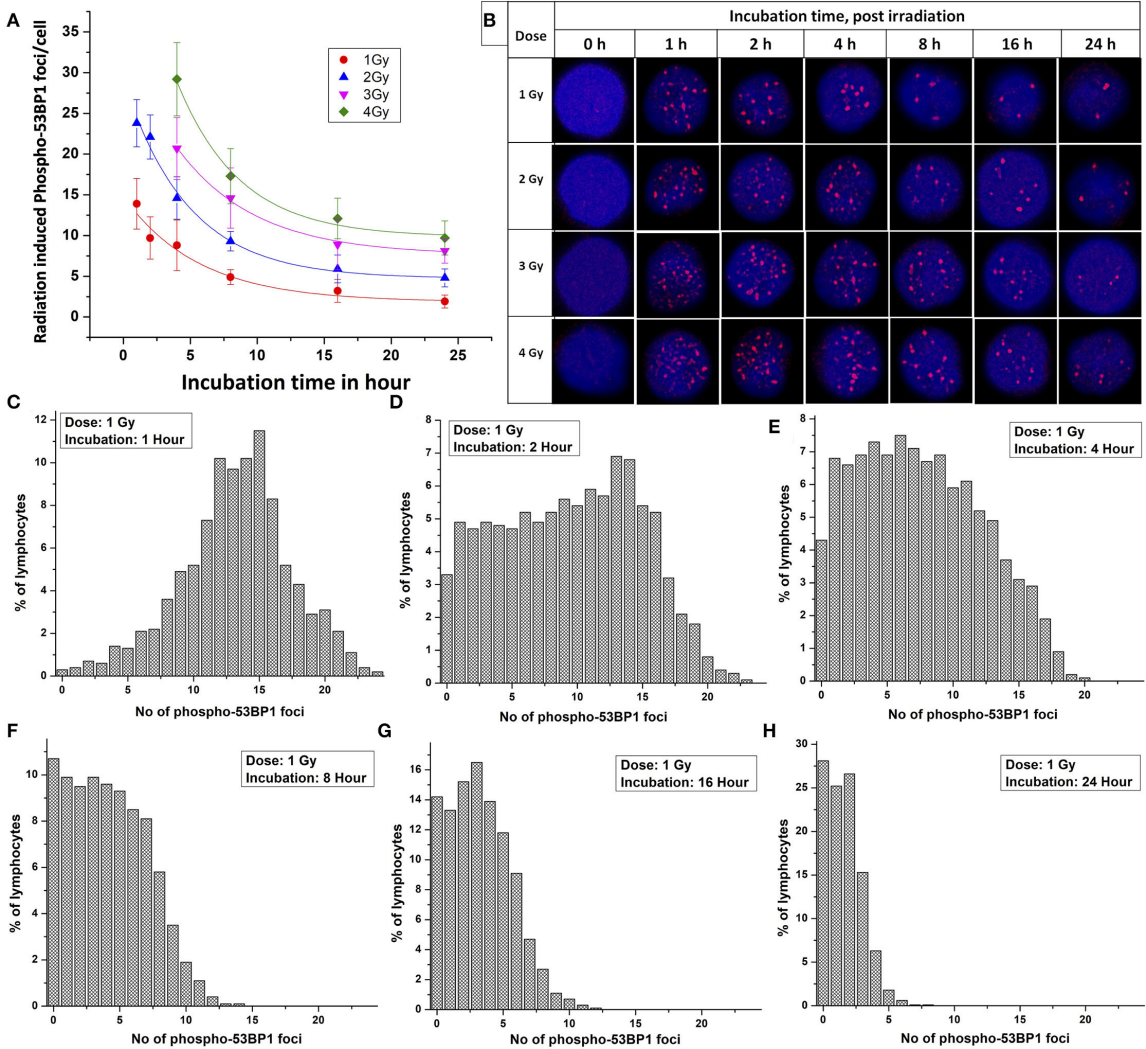
**(A)** Phospho-53BP1 foci, decay kinetics, in the blood sample of five volunteers [two females (with age 22 and 24 y) and three males (with age 25 y, 31 y and 35 y)] after irradiation with 1, 2, 3, and 4 Gy. Error bars represents SEM (*n* = 5 × 3, i.e., five volunteers with triplicate sampling). Number of cells analyzed/scored > 300/dose/time point/volunteer. **(B)** Illustration of the disappearance of phospho-53BP1 foci at 1, 2, 4, 8, 16, and 24 h of incubation after irradiation with 1, 2, 3, and 4 Gy. Dispersion of the number of phospho-53BP1 foci in PBLs irradiated with 1 Gy (cumulative data of all five volunteers) after incubation of **(C)** 1 h **(D)** 2 h **(E)** 4 h **(F)** 8 h **(G)** 16 h, and **(H)** 24 h.

Following acute exposure of 1, 2, 3, and 4 Gy of ^60^C0-γ-rays, phospho-53BP1 foci induction, maturation, and pattern of decay was studied in the lymphocyte of 5 volunteers up to a period of 24 h post-irradiation. In total, 15 samples (five volunteers with triplicate sampling) were fixed and immunofluorescence-stained for the detection of phospho-53BP1 after 0, 1, 2, 4, 8, 16, and 24 h of post-irradiation incubation. Figure 2A illustrates the background-corrected yields of phospho-53BP1 foci with standard deviations for all the doses and post-irradiation incubation time points. As reported earlier (14, 42, 43), phospho-53BP1 foci formation attains maturity at ≤ 1 h of incubation post-irradiation; therefore, data were acquired post 1 h incubation after irradiation, up to 24 h. As illustrated in Figure 2A, the yield of phospho-53BP1 foci was found to be rapidly decreasing with increasing incubation time. Statistical analysis demonstrated that the mean yield of irradiated samples was significantly higher (*p* < 0.05) than the unirradiated sham control, up to 24 h of post-irradiation incubation. The fitted curves corresponding to the experimental data show that after attaining saturation (1 h), the foci decay up to 24 h, followed by a single exponential decay pattern, which followed the mathematical expression Y = A1^*^exp(-x/t1) + Y0, where, A1, Y0, and K (=1/t1) represent the initial value of the exponential function, initial offset of the fit function, and rate of decay of phospho-53BP1 foci, respectively. A1, Y, and Y0 are expressed in phospho-53BP1 foci/cell, k (1/t1) is expressed in h^−1^, and X in h. As illustrated in Table 1, constants A1, Y, and Y0 are dose-dependent, which increase with increasing doses. However, constant K is almost independent of the initial dose delivered (i.e., the rate of decay of phospho-53BP1 foci is almost the same for all the doses). The parameter t1 represents the mean life of foci, which reflects the duration after which the number of foci becomes 1/e or 36% of the initial peak number of foci i.e., foci observed at 1 h of incubation after irradiation. The bar charts, illustrated in Figures 2C–H, show the dispersion of the number of foci in PBLs as a function of time after irradiation (1 Gy). For the data obtained at 1 and 2 h post-irradiation, the dispersion index was found to be close to one. Papworth's *u*-values were −0.89 and 1.54 for 1 and 2 h of incubation post-irradiation, respectively. *U*-values were within the range of −1.96 to + 1.96, indicating that foci distribution followed the Poisson distribution. Data obtained from 4, 8, 16, and 24 h of incubation post-irradiation showed over-dispersion of the foci (*u*-values were higher than 1.96).

**Table 1:** Fitting parameters for phospho-53BP1 foci decay for doses 1, 2, 3, and 4 Gy up to 24 h of incubation after irradiation.

**Dose (Gy)**	Fitting parameters: Foci decay following single exponential decay pattern, mathematically expressed as Y = A1*exp(-x/t1) + Y0		
	**Y0 (foci/cell)**	**A1 (foci/cell)**	**K(=1/t1) (h^**−1**^)**
1	1.66	11.99	5.68
2	5.14	22.99	4.97
3	6.86	24.75	5.76
4	8.54	41.91	5.42

As illustrated in Table 2, the comparable half-lives demonstrate that phospho-53BP1 foci decay kinetics, i.e., DSB repair half-lives, are nearly independent of initial doses delivered. This gives an indication of efficient repair of DSBs even at high doses, and that the decay pattern (DSB-repair) follows a Poisson distribution (up to 2 h for 1 and 2 Gy, and up to 4 h for 3 and 4 Gy, *u*-values were within the range of −1.96 to +1.96).

**Table 2:** Half-lives of phospho-53BP1 foci for doses of 1, 2, 3, and 4 Gy estimated using the formula T1/2 = 0.693*t1.

**S. No**.	**Dose (Gy)**	**Estimated half life (T_**1/2**_) in h**
1	1	3.9
2	2	3.5
3	3	3.9
4	4	3.7

### Dose-Response Curves

**Figure 3:**
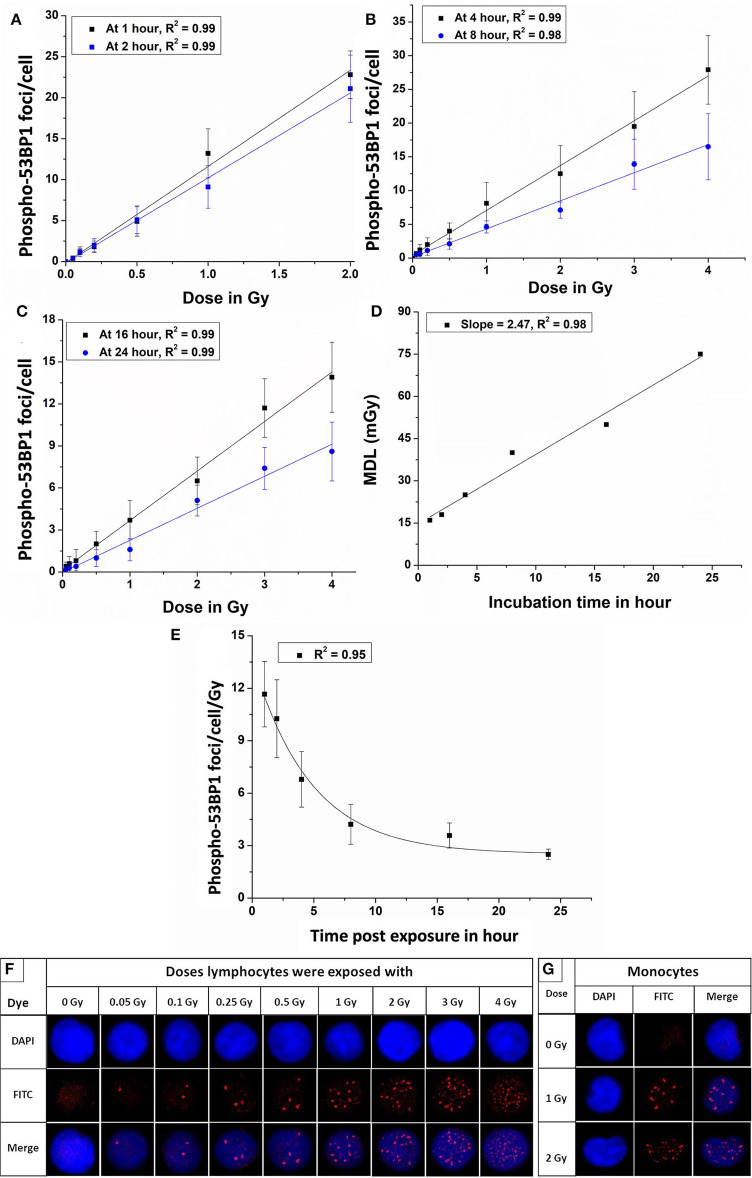
Dose-response curves for ^60^Co-γ-rays-induced-phospho-53BP1 foci formation post-irradiation incubation of **(A)** 1 and 2 h **(B)** 4 and 8 h **(C)** 16 and 24 h. **(D)** Estimated minimum detection limits of the dose-response curves established at 1, 2, 4, 8, 16, and 24 h of incubation, following ISO recommendations (3 sigma of the background frequency) **(E)** Variation of the slope (53BP1 foci/cell/Gy) of the established dose-response curves, generated at 1, 2, 4, 8, 16, and 24 h of incubation, post-irradiation. **(F)** Microscopic images showing an increasing number of phospho-53BP1 foci in the nucleus of PBLs with increasing doses (0 to 2.0 Gy after 1 h and 3 and 4 Gy after 4 h of incubation, post irradiation). **(G)** Representative images of monocytes (kidney-shaped nucleus) with and without 53BP1 foci (these cells were excluded from the scoring of 53BP1 foci).

Dose-response curves were generated for ^60^C0-γ-rays-induced phospho-53BP1 in the PBLs of all 5 volunteers at 1, 2, 4, 8, 16, and 24 h post irradiation. Isolated PBLs were irradiated with 0, 0.05, 0.1, 0.25, 0.5, 1, 2, 3, and 4 Gy in serum-free RPMI medium and incubated in optimum conditions (5% CO_2_, 95% relative humidity, and 37°C temperature). Serum was added immediately after irradiation. Since growth factors are liable to radiation-induced denaturation, the presence of serum during irradiation was avoided. For the dose range 0.05–4 Gy, response curves were generated at 4, 8, 16, and 24 h of incubation, post-irradiation. The results of the experiments are plotted in Figures 3A–F. The regression analysis and data plotting were carried out using software OriginLab 9.65. The data were fitted by a linear model of expression *Y* = α*D* + *C*. Since background frequency was subtracted from all the dose and time point data sets, Y-intercept was kept at zero (C = 0).

The equations for the linear response curves generated, Figures 3A–C, are given below (after 1, 2, 4, 8, 16, and 24 h of incubation, respectively).


(1) $$\begin{array}{rcl} Y_{1} & = & D_{1}*11.65\left(\pm 1.87\right), \end{array}$$

(2) $$\begin{array}{rcl} Y_{2} & = & D_{2}*10.26\left(\pm 2.11\right), \end{array}$$

(3) $$\begin{array}{rcl} Y_{4} & = & D_{4}*6.79\left(\pm 1.02\right), \end{array}$$

(4) $$\begin{array}{rcl} Y_{8} & = & D_{8}*4.22\left(\pm 0.91\right), \end{array}$$

(5) $$\begin{array}{rcl} Y_{16} & = & D_{16}*3.59\left(\pm 0.72\right),\text{ and} \end{array}$$

(6) $$\begin{array}{rcl} Y_{24} & = & D_{24}*2.28\left(\pm 0.18\right). \end{array}$$

A good linear fit with the correlation coefficient (r) ~ 0.98 or better was observed.

The Equations 1–6 are obtained after fitting the experimental data points shown in Figures 3A–C. These equations can be applied for the estimation of unknown doses and has been applied in Section Comparative Evaluation of Established Response Curves by Phospho-53BP1, γH2AX, Dicentric, and Translocations Assays Using Dose-Blinded Samples.

Figure 3D shows the linear fit of the MDLs of the dose-response curves generated at different incubation time points (1–24 h), post-irradiation.

Figure 3E represents a pattern of change of the slopes (phospho-53BP1 foci per cell per Gy) vs. time post-exposure for 6 dose-response curves generated at 1–24 h post-irradiation. The exponential pattern of decay with the decay constant at 0.34 h^−1^ is observed. As illustrated in Figure 3F, the number of foci increased with increasing doses. All sets of data were in agreement with Poisson distribution, and Papworth's *u*-values were found to be ranging from −1.24 to + 0.99. *U*-values were within the range of −1.96 to +1.96, indicating that the foci distribution was followed the Poisson distribution. For the dose range 0.05–2 Gy, induced foci were distinct and non-overlapping throughout the observation period i.e., 1–24 h, post-irradiation. However, overlapping (indistinguishable foci) of foci were observed in the PBLs irradiated with 3 and 4 Gy with incubation of 1 and 2 h. Therefore, samples with higher doses (3 and 4 Gy) were incubated for longer durations (≥4 h) to allow some DSBs to get repaired, and subsequently the number of foci get reduced to the level of distinguishable range (44, 45). Figure 3G represents monocytes (with kidney-shaped nucleus), and these cells were not taken into account for the scoring of 53BP1 foci in any experiment.

### Colocalization of Phospho-53BP1 and γH2AX, DDR Proteins, and Their Correlation

**Figure 4:**
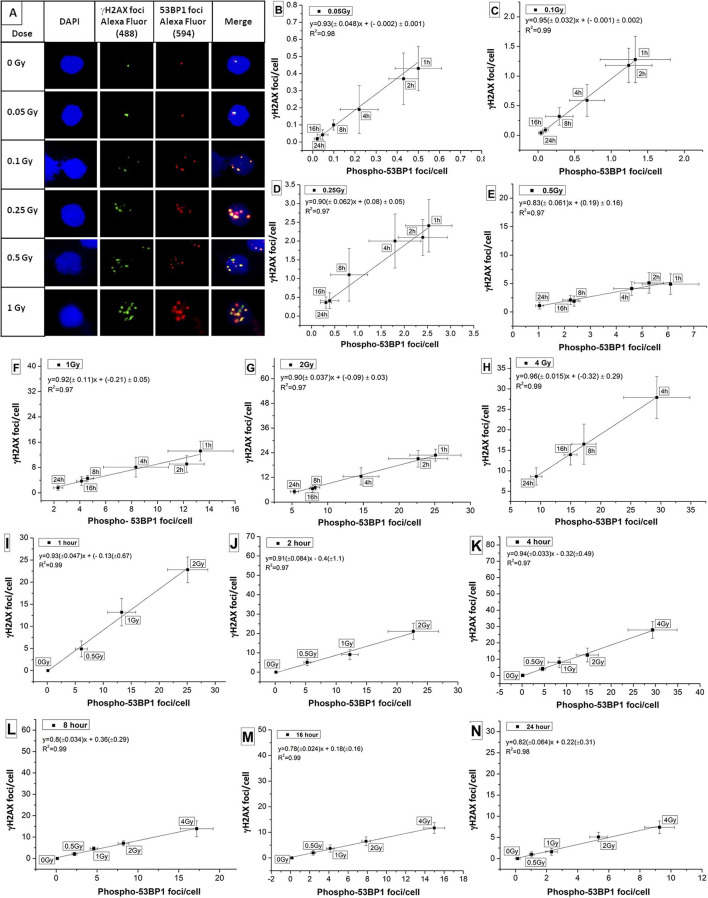
**(A)** Colocalization of phospho-53BP1 and γH2AX foci in the nucleus of human lymphocyte exposed with various doses (at 1 h incubation post irradiation). Correlations established between dose-response curves generated for phospho-53BP1 foci and γH2AX foci (data not shown) at dose points **(B)** 0.05 Gy **(C)** 0.1 Gy **(D)** 0.25 Gy **(E)** 0.5 Gy **(F)** 1 Gy **(G)** 2 Gy **(H)** 4 Gy with varying incubation time points (1–24 h) after irradiation. Correlations were established between dose-response curves generated for phospho-53BP1 foci and γH2AX foci, at **(I)** 1 h **(J)** 2 h **(K)** 4 h **(L)** 8 h **(M)** 16 h and **(N)** 24 h of incubation after irradiation with various doses (0–4 Gy).

Colocalization of candidate marker proteins of DDR, viz, phospho-53BP1 and γH2AX was studied in human PBLs in the dose range of 0–1 Gy. As illustrated in Figure 4A, γH2AX foci were stained with green, phospho-53BP1 foci were stained with red, and merged foci were combination of green + red (localized in close vicinity). It was observed that both kinds of foci (red and green) were localized in the nuclear region of the PBLs. Enumeration of foci indicated that 76–89% phospho-53BP1 and γH2AX foci were in close vicinity, indicating their colocalization, and the rest of the foci were located physically distant from each other, indicating their non-colocalization. Results exhibited that the number of foci colocalized were dependent on dose as well as the time of incubation post-irradiation. Fraction of colocalization was found to be increasing with the increasing dose and the incubation time after irradiation.

Correlation was established for the phospho-53BP1 and γH2AX (data not shown here) at various doses (0.05–4 Gy) and incubation time points (1–24 h). For dose points 0.05, 0.1, 0.25, 0.5, 1, 2, and 4 Gy (at incubation of 1–24 h), the Pearson correlation coefficient (*R*^2^) was found in the range of 0.97 to 0.99 (Figures 4B–H), indicating a strong positive correlation. Similarly, correlations were established at the incubation of 1, 2, 4, 8, 16, and 24 h post-irradiation (0–2 Gy at 1 and 2 h, and 0–4 Gy at 4, 8, 16, and 24 h). Strong positive correlations (correlation coefficients between 0.97 and 0.99) were observed between these two proteins (Figures 4I–N).

These two DSB repair proteins (phospho-53BP1 and γH2AX) are considered almost equally sensitive and quantifiable markers of DSB detection. Together, they can give dual confirmation of dose estimation in relation to emergency biodosimetry and clinical investigations.

### Comparative Evaluation of Established Response Curves by Phospho-53BP1, γH2AX, Dicentric, and Translocations Assays Using Dose-Blinded Samples

**Figure 5:**
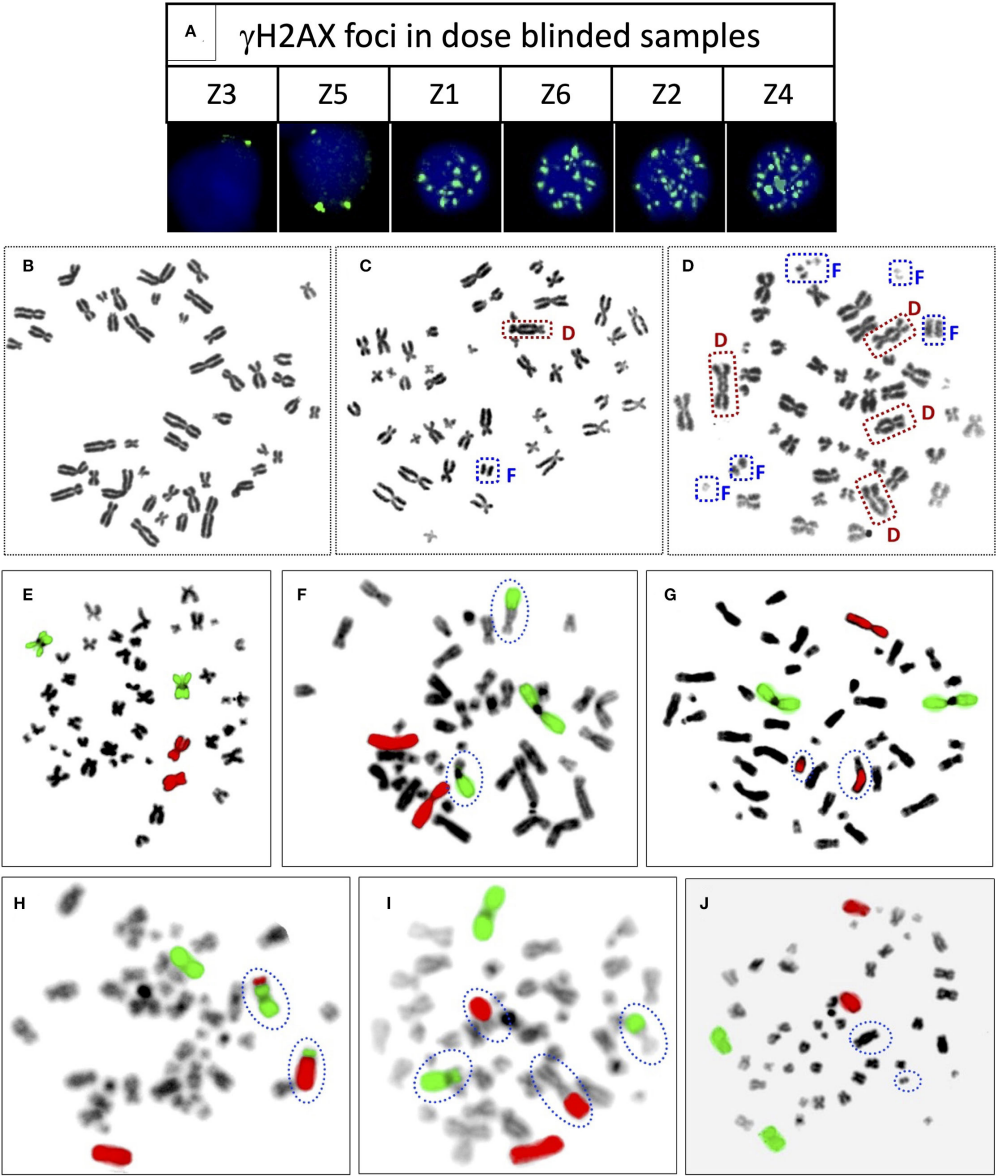
**(A)** Representative PBLs of dose blinded samples Z3 (1 h), Z5 (1 h), Z1 (1 h), Z6 (1 h), Z2 (4 h), and Z4 (4 h) with their respective number of γH2AX foci. Representative, Giemsa stained metaphase spreads with **(B)** no dicentric **(C)** one dicentric with one fragment **(D)** three dicentrics and one tricentric with five fragments. Representative metaphase spreads processed for FISH, chromosome pairs 1 and 2 hybridized with whole chromosome paint probes tagged with green and red fluorophores, respectively. Metaphase with **(E)** no aberration **(F)** one RT between green and black chromosomes **(G)** one RT between red and black chromosomes **(H)** one RT between green and red chromosomes **(I)** two RT, between green and black, and red and black **(J)** one dicentric, such metaphases were excluded from the scoring.

**Figure 6:**
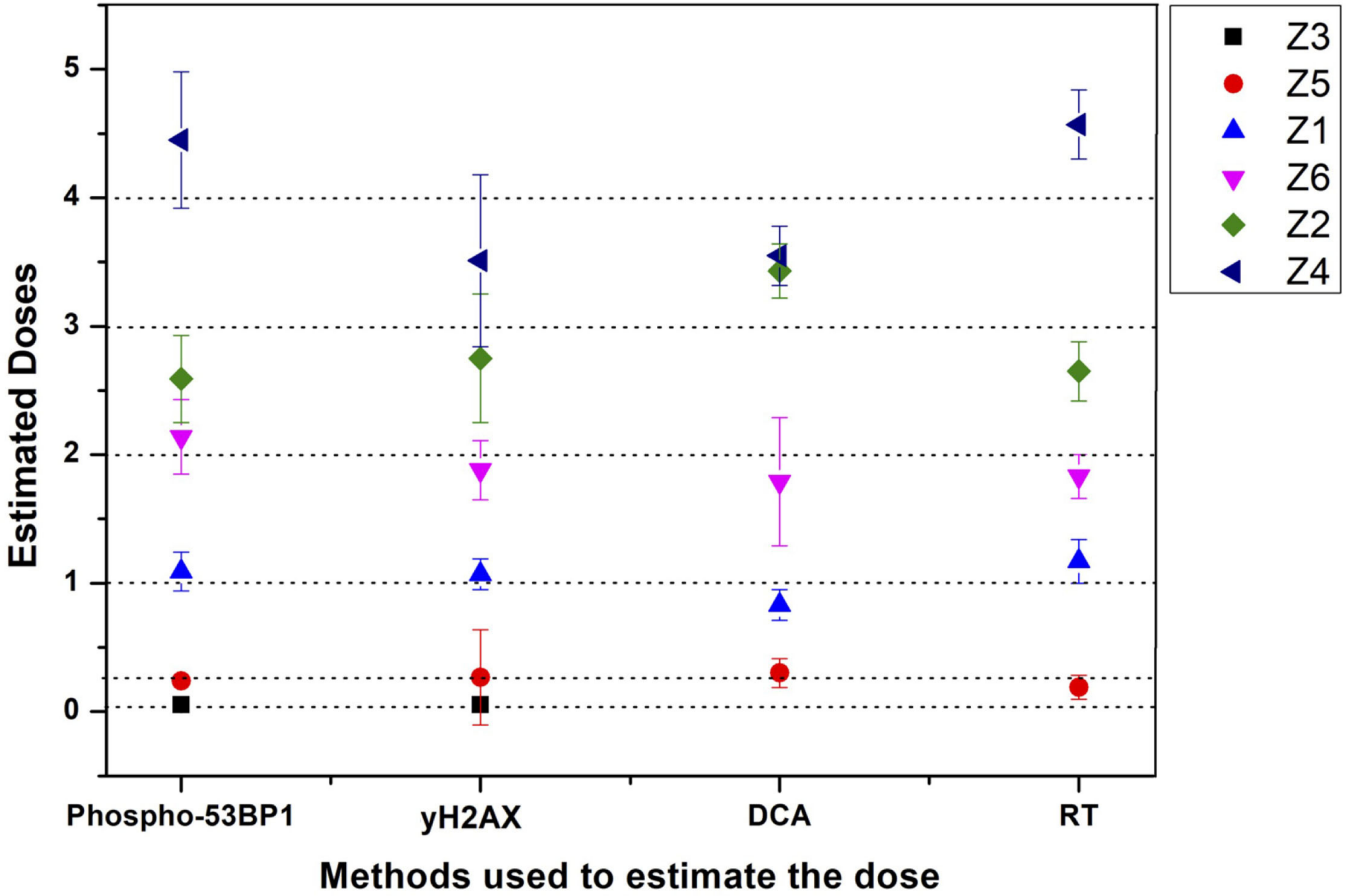
Graphical illustration of the estimated doses of blinded samples (Z1–Z6) by immunofluorescence (phospho-53BP1 and γH2AX) and cytogenetic (dicentric and reciprocal translocation) assays.

Established dose-response curves were validated with 6 dose-blinded samples and comparatively evaluated with cytogenetic and γH2AX immunofluorescence assays (Figures 5, 6, Table 3). Keeping in mind, the possibility of overlapping of foci (phospho-53BP1 and γH2AX) at higher doses, all blinded samples were fixed and processed at two different incubation time points (i.e., 1 and 4 h) post-irradiations. More than 300 cells were analyzed to score phospho-53BP1 and γH2AX foci independently in each blinded sample.

**Table 3:** Comparative evaluation of established response curve (phospho-53BP1) by estimating biological doses of 6 dose blinded samples by phospho-53BP1, γH2AX, dicentric, and translocation assays.

**S. N**.	**Dose delivered (Gy)**	**Dose estimation by immunofluorescence assays**				**Dose estimation by cytogenetic assays**			
		**53BP1**		**γH2AX**		**Dicentrics**		**Reciprocal translocations**	
		**Estimated dose (Gy)**	**% variation**	**Estimated dose (Gy)**	**% variation**	**Estimated dose (Gy)**	**% variation**	**Estimated Dose Gy)**	**% variation**
Z1	1.0	1.09 {95% CI: 0.94–1.24}	+ 9	1.07 {95% CI: 0.95–1.19}	+7	0.829 {95% CI: 0.709–0.956}	−17.1	1.17{95% CI: 0.999–1.34}	+ 16
Z2	3.0	2.59 {95% CI: 2.25–2.93}	−13.7	2.75 {95% CI: 2.25–3.25}	−10.3	3.43 {95% CI: 3.22–3.64}	+14.2	2.65 {95% CI: 2.42–2.89}	−11.7
Z3	0.05	0.053 {95% CI: 0.046–0.06}	+6	0.054 {95% CI: 0.048–0.06}	+8	In the background range	–	In the background range	–
Z4	4.0	4.45 {95% CI: 3.92–4.98}	+11.3	3.51 {95% CI: 2.84–4.18}	−12.2	3.55 {95% CI: 3.32–3.78}	−11.3	4.57 {95% CI: 4.30–4.84}	+14.3
Z5	0.25	0.24 {95% CI: 0.22–0.26}	−4.1	0.267 {95% CI: 0.235–0.299}	+7.2	0.301 {95% CI: 0.189–0.431}	+20.3	0.189 {95% CI: 0.096–0.397}	−24
Z6	2.0	2.14 {95% CI: 1.85–2.29}	+7	1.88 {95% CI: 1.65–2.11}	−6	1.79 {95% CI: 1.29–2.93}	−10.5	1.83 {95% CI:1.66–1.99}	−8.5

*Doses for the blind samples Z1, Z3, Z5, Z6, and Z2, Z4 were estimated by using 1 and 4 h calibration curves, respectively (for both phospho-53BP1 and γH2AX). Cytogenetic (dicentric and translocation) biological doses were estimated following in-house established calibration curves, with coefficients, alpha 2.7 × 10^−2^ Gy^−1^ and beta 6.5 × 10^−2^ Gy^−2^ and alpha 0.90 × 10^−2^ Gy^−1^ and beta 3.58 × 10^−2^ Gy^−2^ for dicentrics and translocations, respectively. Dose of sample Z-3 (0.05 Gy) was not estimated by cytogenetic tools (dicentric and translocation), as it was below the detection limits of both the assays*.

It was observed that foci were nonoverlapping and distinguishable in samples Z1, Z3, Z5, and Z6 (Figure 5A); however in samples Z2 and Z4, the foci were overlapping, numerous in number, and indistinguishable at 1 h of incubation post-irradiation. Therefore, the number of foci was quantified in samples Z1, Z3, Z5, and Z6 at 1 h of incubation, and the doses were estimated by applying coefficients generated at the 1 h dose-response curve. Incubation in optimum conditions leading to DSB repair and subsequent loss of foci allowed us to quantify the number of foci at 4 h of incubation in samples Z2 and Z4 (Figure 5A), and doses were estimated by applying coefficients generated at 4 h dose–response curve. Results presented in Table 3 show that estimated doses were well within ± 14% of the actual doses delivered.

All dose blinded samples were further processed by gold standard dicentric and reciprocal translocation assays as well. More than 500 metaphases were analyzed to score dicentrics (Figures 5B–D) and reciprocal translocations (Figures 5E–J), independently, following ISO and IAEA scoring criteria (9, 37, 41). Doses were estimated by using in-house established calibration curve for dicentrics with coefficients alpha of 2.7 × 10^−2^ Gy^−1^ and beta of 6.5 × 10^−2^ Gy^−2^ (36), and for reciprocal translocations with coefficients alpha of 0.90 × 10^−2^ Gy^−1^ and beta of 3.58 × 10^−2^Gy^−2^ using chromosomal aberrations calculation software (CABAS). For sample Z3, yields of dicentrics and translocations were found in the range of variation (3 sigma) of the background frequency; hence it can be considered that Z3 was below the detection limits for both the assays. For the samples Z1–Z6 (except Z3), estimated doses were well within ±25% of the actual doses delivered. Diagrammatic illustration of blind-folded doses estimated by cytogenetic and immunofluorescence (phospho-53BP1 and γH2AX) assays is depicted in Figure 6.

## Discussion

The variation in the background frequency of phospho-53BP1 foci can serve as a tool for the establishment of MDL of the assay. Variation in the mean background frequency of phospho-53BP1 was measured in the blood sample of all five volunteers (V1–V5). As per ISO recommendations for the estimation of MDL of the assay, 3σ of the background frequency (0.12 ± 0.057 foci/cell) was estimated and it was found to be 0.171 foci/cell and their corresponding dose was ~15 mGy (applying dose–response curve equation generated at 1 h of incubation post-irradiation).

Rasche et al. reported the background level of phospho-53BP1 in 26 local healthy control volunteers of Berlin. The median number of foci observed was 0.04, ranging from 0 to 0.49 (46). In another study involving 3 healthy test volunteers conducted in the Department of Nuclear Medicine of the University Hospital Würzburg, Würzburg, Germany, the mean background level of phospho-53BP1 was 0.17 ± 0.04 foci/cell, ranging from 0.10 to 0.25 foci/cell (47). Recently, a study conducted in Bavaria, Germany has shown the baseline mean frequency in unirradiated sham control lymphocytes, which was 0.14 ± 0.04 (mean ± SE) (48). Overall, the observed background level of phospho-53BP1, in the reviewed population ranged from 0 to 0.49 foci/cell. The background frequency we observed in this study was within the range of the background frequency reported in the reviewed populations mentioned above. Background frequency of phospho-53BP1 is attributed to various endogenous and exogenous factors, which are known to induce reactive oxygen species (ROS) and subsequent DSBs in the cells (49–51). Some other cellular processes like cell-division and cell-differentiation can also induce DSBs (52, 53).

Earlier reports have shown that phospho-53BP1 decay kinetics follow single, as well as biexponential decay pattern (54–56). In this study, data were obtained up to 24 h, post-exposure, and it was observed that phospho-53BP1 foci decay followed a single exponential decay pattern (Figure 2A). On analysis of the published data, it was observed that fitting of the data obtained from longer incubation periods follows a biexponential decay pattern. However, fitting of the data obtained from comparatively shorter incubation period follows single-exponential decay pattern (54, 57–59). Biexponential decay pattern consists of an initial fast decay component (t_1/2fast_, shorter half-life) followed by a slow decay component (t_1/2slow_, comparatively longer half-life) (60, 61). Though, single exponential decay consists of only one decay component which falls in between the fast and slow decay components. In this study calculated t_1/2_ was found to be 3.5–3.9 h (for different doses, Table 2). Fast decay component corresponds to faster repair of simple DSBs and slow decay component corresponds to complex DSB repair, which is relatively slow and time-consuming. In case of single exponential decay pattern, t_1/2_ is relatively longer than t_1/2fast_ and shorter than t_1/2slow_, which corresponds to the repair of simple as well as complex DSBs.

Ionizing radiation is known to induce heterogeneous DSBs in DNA, and some DSBs are associated with other DNA lesions, resulting in the formation of complex DSBs, which are difficult and time-consuming to get repaired, such foci persist for longer durations (Figure 2B) (62). Simple DSBs get repaired in a short span of time (within a few hours), though, clustered DSBs warrant adequate time (several hours to days depending upon the complexity in DNA damage) (63). Sometimes clustered DSBs remain unrepaired and can lead to apoptotic cell death (64). Graphical illustration for dispersion of phospho-53BP1 foci for 1 Gy is shown in Figures 2C–H. Due to the faster repair of simple DSBs, the distribution of cells with a higher number of foci shifted toward cells with a lower number of foci with increasing incubation time. Foci persisting up to 24 h of incubation indicate DSBs, which are difficult and time-consuming to get repaired (Figure 2B) (28).

Considering the possibilities of delay in blood sampling, following any unplanned radiation exposures or radiological emergencies, dose-response curves were generated up to a period of 24 h after radiation exposure. The time of exposure should be known to select and apply an appropriate dose-response curve to estimate the unknown dose. Following ISO recommendations (3 sigma, variation of the background frequency), MDLs were established for all 6 dose-response curves (Figure 3D) and it was found to be ~15, ~18, ~25, ~40, ~50, and ~75 mGy for the dose-response curves generated at 1, 2, 4, 8, 16, and 24 h (post-irradiation), respectively. Due to the inherent nature of DSB-repair of living cells, the number of phospho-53BP1 foci/cell decreases with increasing incubation time, leading to an increase in MDLs (65–67). MDLs were found to be increasing linearly with increasing post-irradiation incubation time with a slope of 2.47 mGy/h (Figure 3D). These findings can help to estimate the MDL of the assay at any given time of incubation, post-irradiation.

Currently, the colocalization of phospho-53BP1 and γH2AX foci, is considered as the most reliable and dependable marker of DSB enumeration (68, 69). Tagging together, these two proteins give dual confirmation (better confidence) for the quantification of DSB repair foci (phospho-53BP1 + γH2AX) and subsequent reconstruction of the dose. Figure 4A illustrated that up to 89% phospho-53BP1 and γH2AX foci were colocalized, though the rest of the foci were physically apart from each other. The difference in the number of phospho-53BP1 and γH2AX foci induced, at any given dose and incubation time (post irradiation) point, maybe the reason behind it (44, 45, 54). More exploration is warranted to understand the limitation of these candidate protein markers and kinetics of their colocalization (co-persistence) after DSB formation (or radiation exposure) (70–72).

Comparative evaluations of established response curves were made with immunofluorescence (phospho-53BP1 and γH2AX foci) and cytogenetic assays by estimating doses of dose blinded samples (Z1–Z6), illustrated in Table 3 and Figure 6. For the lower doses, i.e., samples Z1, Z3, Z5, and Z6 (≤ 2 Gy), estimated doses by immunofluorescence (i.e., by phospho-53BP1 and γH2AX) assays were reasonably close within 9% from the doses delivered. However, for higher doses i.e., samples Z2 and Z4 (≥3 Gy), the estimated doses were still close but showed variations within 14.3% of the actual doses delivered. These findings indicate that at lower doses (i.e., ≤ 2 Gy), immunofluorescence (γH2AX and phospho-53BP1) assays made closer estimates in comparison with higher doses i.e., ≥3 Gy.

The lowest blinded dose Z3 (0.05 Gy) was found to be below the detection limits of both the cytogenetic assays (dicentrics and translocations). As may be seen in Table 3, the estimated dose for the sample Z5 (0.25 Gy), showed substantial variation, within 24%, for dicentrics and translocations. In contrast, for the higher doses, cytogenetic assays made closer estimates (14.3 % variation) in comparison to lower doses (24% variation). For the higher doses (i.e., ≥3 Gy), both cytogenetic and immunofluorescence (γH2AX and phospho-53BP1) assays made comparable and closer estimates, which was within the variation of 14.3%.

Doses estimated for all the blinded samples (Z1–Z6), by all the implied assays were within the variation of −4.1 to −24% of the doses delivered. For the lower doses i.e., Z1, Z3, Z5, and Z6 immunofluorescence (γH2AX and phospho-53BP1) assays gave comparatively closer estimates (with maximum variation of + 9 %) in comparison to cytogenetic assays (−24% variation) and for the higher doses i.e., Z2 and Z4, cytogenetic and immunofluorescence (γH2AX and phospho-53BP1) assays made comparable close estimates, with the maximum variation of +14.3 and −13.7%, respectively.

All experiments of the present work were carried out with human peripheral blood mononuclear cells (PBMCs), isolated by Histopaque-1077, which majorly consist of lymphocytes (70–90%) and monocytes (10–20%) (73). In this study, only lymphocytes, characterized by uniform-round nucleus (74) were taken into account (Figures 1B, 2B, 3F). Monocytes, characterized by, indented-kidney shaped nucleus (75) (Figure 3G) were excluded from the scoring of phospho-53BP1 foci. In a glance, it was observed that the number of phospho-53BP1 foci in monocytes was comparable with the number of foci present in lymphocytes. Recently, Heylmann et al. reported that monocytes exhibit less radio-sensitivity than lymphocytes and the repair process progresses at a comparatively low pace, indicating long persistence of such DSB repair foci (76). It would be interesting to further explore the number and persistence of DSB repair foci in monocytes and its suitability for rapid biodosimetry tool.

In spite of being sensitive and a reliable marker, certain inherent limitations of these DDR proteins cannot be avoided. With the repair of DSBs, these proteins get dephosphorylated and the foci disappear with time, therefore post-exposure sample collection and knowing the proper chronology of exposure is essential, which is difficult in the case of radiological emergencies though possible in case of planned exposures (medical and occupational exposures) (44, 77, 78). The application window of the assay is shorter (maximum, 1–4 days) in comparison to cytogenetic markers that persist from months to decades (6, 27). MDL of the assay increases with the increasing time of blood collection after exposure (due to DSB repair), which cannot be circumvented (28, 45). After the collection of blood, the repair process can be slowed down by storing the sample in cold conditions (79, 80). MDLs of the cytogenetic assays remain constant over the application time window.

## Conclusion

In this study, dose-response curves were established for ^60^C0-γ-rays-induced phospho-53BP1 for the dose range 0.05–4 Gy at 1, 2, 4, 8, 16, and 24 h, post-irradiation. These dose-response curves can be employed for rapid biodosimetry of radiological emergencies and various planned medical exposures when the chronology of exposures is known. Once the blood sample is obtained in the laboratory, the assay can be performed within 3–4 h and the dose can be estimated by applying coefficients of an appropriate calibration curve. With lower MDLs relevant in the range of doses involved in many therapeutic and diagnostic applications, γH2AX and phospho-53BP1 foci assays can serve as a handy tool. Comparative evaluations of phospho-53BP1 were made with γH2AX foci, dicentrics, and reciprocal translocation assays, and it was observed that at lower dose ranges (0.05–2 Gy) immunofluorescence assay (phospho-53BP1 and γH2AX) gave closer estimates than cytogenetic (DCA and RT) assays. However, at higher doses (3 and 4 Gy) all 4 assays gave comparably closer estimates.

The biodosimetry lab of BARC has established phospho-53BP1 along with other standard immunofluorescence and cytogenetic assays, such as γH2AX, DCA, translocation, micronucleus, etc. As per IAEA recommendations, together, all these assays and their in-house established dose-response curves can serve as tools for multi-parametric biological dose estimations to strengthen the statistical confidence.

## Data Availability Statement

The raw data supporting the conclusions of this article will be made available by the authors, without undue reservation.

## Ethics Statement

The studies involving human participants were reviewed and approved by Institutional Ethical Committee (Bhabha Atomic Research Center, Medical Division). The patients/participants provided their written informed consent to participate in this study.

## Author Contributions

All authors listed have made a substantial contribution in designing the project, execution of experiments, generation and analysis of the data, and writing of the manuscript.

## Funding

This work was supported by Bhabha Atomic Research Center, Mumbai, India (host institute) and external funding was not involved.

## Conflict of Interest

The authors declare that the research was conducted in the absence of any commercial or financial relationships that could be construed as a potential conflict of interest.

## Publisher's Note

All claims expressed in this article are solely those of the authors and do not necessarily represent those of their affiliated organizations, or those of the publisher, the editors and the reviewers. Any product that may be evaluated in this article, or claim that may be made by its manufacturer, is not guaranteed or endorsed by the publisher.
